# Nurses' Attitudes in Adolescent Oncology Care: Supporting Adolescents While Navigating Emotional Challenges and Fostering Mutual Subjectivity

**DOI:** 10.7759/cureus.81049

**Published:** 2025-03-23

**Authors:** Michiko Nambu, Mari Matsuoka

**Affiliations:** 1 Department of Child Health Nursing, Mie University Graduate School of Medicine, Tsu, JPN

**Keywords:** adolescent cancer nursing, adolescent oncology, nurses’ attitudes, nursing presence, young adult oncology

## Abstract

Background: In Japan, nurses often find it particularly difficult to communicate with adolescents with cancer due to limited experience in caring for this age group and their unique needs. Conversely, adolescents with cancer perceive the presence of nurses as meaningful in their interactions and essential in helping them rebuild their identities. This study aimed to describe the attitudes of nurses who cared for adolescents with cancer in their daily practice and encountered challenges during these interactions.

Methods: This qualitative descriptive study employed semi-structured interviews. Verbatim transcripts were created from the recorded data, which were analyzed using a thematic analysis approach.

Results: A questionnaire and semi-structured interviews were conducted with 10 nurses who had five or more years of experience in adolescent cancer nursing and worked in wards. The analysis identified five categories of nurses' attitudes: "engaging with adolescents with cancer based on their characteristics," "having expertise as a nurse," "being with adolescents while managing one's own emotional wavering," "caring for adolescents by treating them as a person and valuing their subjectivity," and "experiencing one's own subjectivity."

Conclusion: In adolescent cancer nursing, where nurses tend to experience challenges, it is important to engage with adolescents without rushing to provide answers, to separate oneself from adolescents, and to be present with them while managing emotional wavering. The attitudes of nurses who cared for adolescents with cancer were interpreted as embodying the essence of nursing presence. Further research should incorporate patients' perspectives on nursing presence to help alleviate nurses' sense of difficulty in these interactions.

## Introduction

In Japan, adolescent and young adult (AYA) patients with cancer are defined as those aged 15-39 years. They face inadequate improvements in treatment outcomes, are of reproductive age, and are ineligible for long-term care insurance [1]. The unique needs of AYAs with cancer from their developmental stages and diverse challenges, including financial burdens, disruptions to education and work, relationships, family planning, and physical and mental health, are often insufficiently addressed [2]. Additionally, the small patient population and diverse cancer profiles hinder medical personnel from gaining adequate experience in providing tailored support. Therefore, efforts to address these gaps have been introduced through policy measures [3]. However, only a few facilities in Japan have wards dedicated to AYAs with cancer, and they are typically admitted to adult or pediatric wards, where appropriate support remains limited.

Healthcare professionals face great challenges and consider adolescents among the hardest patients with whom to communicate [4]. These challenges include providing developmentally appropriate support, establishing trusting relationships despite AYAs' immature psychological development, and managing communication-related anxiety and confusion [4,5]. Adolescents (defined in this study as those aged 15-19 years) face particular difficulties in building relationships as they navigate the transition between independence and dependence, a hallmark of this developmental stage [6]. A prior Japanese study reported that nurses experienced heightened challenges in caring for cancer patients aged 15-24 years due to communication difficulties [7].

Conversely, AYAs with cancer have expressed that they feel most at ease when healthcare providers establish meaningful connections and address their needs [5,8]. These interactions typically occur during routine care rather than specific moments, such as end-of-life discussions or treatment planning. AYAs perceive nurses as "being with them," a relational dynamic that plays an important role in the helping relationship [9] and supports the reconstruction of their identities [10].

Despite these insights, the experiences, feelings, and attitudes of nurses toward supporting AYAs with cancer, especially adolescents, remain underexplored. Daily interactions between nurses and adolescent patients are pivotal, given that adolescents are in the process of identity formation and often experience emotional instability. Such interactions can also provide a foundation for care in more critical contexts, such as end-of-life phases. Additionally, the average hospital stay in Japan is longer than in many other countries [11], leading to prolonged interactions between nurses and inpatients. This study aimed to describe the attitudes of nurses toward adolescents with cancer during daily care and to provide recommendations for improving cancer nursing practices for this population. Addressing these challenges is essential, as opportunities for healthcare providers to develop relevant experience are limited. Clarifying these attitudes may improve nursing practices that enable more effective, individualized care for adolescents with cancer and inform training programs for nurses caring for adolescents with cancer.

In this study, nurses' attitudes were defined as nurses' subjective feelings and thoughts regarding their daily interactions with adolescents with cancer, including their approach to providing care and support.

## Materials and methods

Design and participants

To clarify the nurses' own experiences, we chose to conduct a qualitative study by interviewing nurses. The study team recruited nurses working in wards where adolescents with cancer aged 15-19 years were admitted and who had at least five years of experience in adolescent cancer nursing. Adolescents were defined as those aged 15-19 years, consistent with Erikson's psychosocial development theory [12], which encompasses the transition from pediatric to adult care domains in Japan.

Data collection

Participants were recruited using two methods: (1) requests for cooperation sent to the nursing departments of facilities certified as AYA support team model facilities or children's cancer center hospitals, and (2) snowball sampling by the researchers. Hospitals with AYA support teams were identified using the National AYA Cancer Support Team Network on Japan's website. The primary researcher (M.N.) mailed the survey request form to these hospitals and utilized purposeful sampling to recruit participants.

In the first method, at the participating facilities, the head nurse of the ward where adolescents were admitted distributed the survey request to the staff who met the inclusion criteria. In the second method, we sent the survey request to acquaintances of the research team or someone referred to the research team by a participant who had already been interviewed. Semi-structured interviews were conducted with volunteering participants. An interview guide developed by the authors explored the nurses' feelings and thoughts about daily interactions with adolescents with cancer, as well as their approaches to providing support. The interview guide was developed based on previous studies of nurses' experiences and refined based on advice from two nurses: one pediatric nurse specialist and one oncology nurse specialist with extensive clinical experience. A pre-interview with an oncology nurse specialist was conducted to obtain content validity of the guide, and the feedback from the participants of the pre-interview further refined the guide. The final interview guide is presented in Table 1.

**Table 1:** Interview guide

Interview guide
1. What do you consider important, conscious, or mindful in your daily interactions with people of this generation? Why do you think this way? How did you come to think this way, and what triggers you to think this way?
2. What do you feel or think about in your daily interactions with people of this generation (e.g., daily responsibilities)? Why do you feel or think this way? When do you feel or think about these things? What actions do you take, and why?
3. In your daily interactions with people of this generation, do you feel troubled, lost, or face difficulties? When do these moments occur, and why? What do you do during these times, and why?
4. In your daily interactions with people of this generation, do you sometimes experience positive feelings (e.g., enjoyment or fun)? When do these moments occur, and why? What do you do during these times, and why?
5. Reflecting on this interview, please share any additional thoughts or feelings about your everyday work with adolescents.

Before the interviews, a questionnaire was administered to collect participants' demographic and professional attributes. This questionnaire was identified at the beginning of the interview. Considering the impact of the COVID-19 pandemic, both web-based and face-to-face interviews were offered, allowing participants to select their preferred method and location. In web-based interviews, the researcher participated from a private room, and the URL for the web interview was password-protected to prevent others from entering. Data were collected between June 2022 and September 2022. The first author (M.N.) conducted all interviews. Each interview was scheduled to last 60-90 minutes. The target sample size was approximately 10 cases, referring to previous studies in Japan, and recruitment continued until data saturation was confirmed. Participants were not provided with any incentives for their participation.

Data analysis

Using a qualitative descriptive research approach, the interviews were audio-recorded, transcribed verbatim, and anonymized. Data were analyzed through thematic analysis following Braun and Clarke's six-step framework [13]: familiarization with data, generating initial codes, identifying themes, revising themes, defining and naming themes, and report writing. The analysis was conducted separately by two researchers. In the first step, the first (M.N.) and second (M.M.) authors thoroughly read the interview transcripts. In the second step, they generated codes through repeated readings of the transcripts. Manual data handling was employed. During the third and fourth steps, the two researchers regularly discussed and compared research materials and coding, refining and consolidating concepts and themes until mutual agreement was achieved. If mismatches occur during this process, we discussed until a consensus was reached. After analyzing eight cases and identifying themes, two additional cases were analyzed to confirm that no new concepts or themes emerged, and theoretical saturation was reached. In the final step, only the final results were translated from Japanese to English for submission to an international journal.

Ethical consideration

This study was approved by the Institutional Review Board of Kyoto University Graduate School and Faculty of Medicine, the institution to which the authors were affiliated at the time of the study (approval number: R3404). Before the interviews, the participants were informed about the study's purpose, the voluntary nature of their participation, the data collection methods, the confidentiality of data, and the measures taken to ensure anonymity. They were also required to sign an informed consent form.

## Results

Table 2 presents the demographic characteristics of the participants. A total of 10 nurses participated in a single interview, which lasted an average of 68.3 minutes (range: 58-93 minutes). The mean age of the participants was 39.7 years. The mean number of years of nursing experience was 16.2, while the mean number of years of adolescent cancer nursing experience was 11.4. All participants were women.

**Table 2:** Participants' attributes and background *Pediatric Hematology and Oncology; **Certified Nurse Specialist in Japan

Participant	Age (years)	Sex	Hospital wards	Years of nursing experience	Adolescent cancer nursing years of experience	Involved in the annual number of adolescents with cancer	Qualifications
A	30s	Woman	PHO*	11 years	11 years	10–15 cases	Oncol. Nursing CNS**
B	40s	Woman	PHO	19 years	10 years	>15 cases	None
C	30s	Woman	Ped + Adult Hematology	17 years	15 years	1-2 cases	Ped. Nursing CNS
D	40s	Woman	Ped + Adult Hematology	13 years	5 years	3–5 cases	None
E	30s	Woman	Ped + Adult Medicine	13 years	13 years	>15 cases	None
F	50s	Woman	Pediatrics	32 years	10 years	3–5 cases	None
G	40s	Woman	Pediatrics	16 years	9 years	3–5 cases	Ped. Nursing CNS
H	20s	Woman	Pediatrics	8 years	8 years	6–10 cases	Ped. Oncol. Consultant
I	40s	Woman	PHO	16 years	16 years	>15 cases	Ped. Nursing CNS
J	40s	Woman	Pediatrics	17 years	17 years	6–10 cases	Ped. Nursing CNS

Data were categorized into five themes and 12 subthemes (Table 3).

**Table 3:** Attitudes of nurses caring for adolescents with cancer

Theme	Subtheme
Engaging with adolescents with cancer based on their characteristics	Engaging with an eye to the future
	Communicating with a sense of distance
Having expertise as a nurse	Involving other professions
	Interacting with them as a nurse
Being with adolescents while managing one’s own emotional wavering	Emotions tend to move easily in relation to this generation
	Seeking to make my involvement as good as it can be
Caring for adolescents by treating them as individuals and valuing their subjectivity	Valuing the adolescents’ subjectivity
	Being aware of our relationship as individuals, not just as patient and nurse
	Being aware of our interactions with them on a daily basis
Experiencing one's own subjectivity	Reflecting on my own feelings as an individual
	Feeling satisfaction in supporting their growth
	Experiencing ease of involvement by recognizing the other person as a subject

Nurses who interacted with adolescents with cancer were (1) engaging with adolescents with cancer based on their characteristics, (2) having expertise as a nurse, (3) being with adolescents while managing one's own emotional wavering, (4) caring for adolescents by treating them as a person and valuing their subjectivity, and (5) experiencing one's own subjectivity despite their difficulties.

Engaging with adolescents with cancer based on their characteristics

This theme captures nurses' perspectives regarding adolescents with cancer in nursing practice. They provided care based on the unique characteristics of adolescents with cancer, such as their future after treatment and the sensitivity required in communication. The following two subthemes constitute this theme.

Engaging With an Eye to the Future

Nurses cared not only about the successful completion of treatment but also about its future impact and adolescents' lives post-treatment. "The treatment experience helped them grow, and I hope it will help them later" (Ms. D).

Communicating With a Sense of Distance

Nurses understood the sensitivity of communication with adolescents and assessed the appropriate level of closeness. "I do not keep a distance from them, but I am careful not to be too aggressive" (Ms. E).

Having expertise as a nurse

Nurses described their professional expertise and how they developed their nursing identity. This theme included two subthemes.

Involving Other Professions

Nurses collaborated with other professionals to provide comprehensive care for adolescents rather than addressing their needs alone. "I think it would be better to have as many people from different professions as possible come in and keep as many people involved as possible so that they can say something to someone when they want to say" (Ms. E).

Interacting With Them as a Nurse

Nurses distinguish their personal emotions from their professional responsibilities to maintain their role. "Do not connect my feelings with my work as a nurse" (Ms. I).

Being with adolescents while managing one's own emotional wavering

Although nurses experienced challenges and emotional wavering while working with adolescents, they managed their emotions to remain supportive. The following two subthemes constitute this theme.

Emotions Tend to Move Easily in Relation to This Generation

Nurses noted that they were more emotionally affected when caring for adolescents with cancer compared to other age groups, as adolescence is marked by significant life events. "This is a unique time in the lives of this generation, a time often referred to as 'the event of life,' … so it makes me more passionate" (Ms. F).

Seeking to Make My Involvement As Good as It Can Be

Nurses grappled with how to provide better care while navigating the difficulties of their work. "I am also searching for ways to make it seem like it was a good thing" (Ms. E).

Caring for adolescents by treating them as individuals and valuing their subjectivity

In working with adolescents with cancer, nurses treated them as persons rather than patients and prioritized their subjectivity, which they were still developing. The following three subthemes constitute this theme.

Valuing the Adolescents' Subjectivity

Nurses based their care on the adolescents' thoughts and feelings rather than the opinions of parents or medical staff. "What they want to do is the initiative over there, not me (Ms. B).

Being Aware of Our Relationship as Individuals, Not Just as Patient and Nurse

Nurses valued their relationships with adolescents beyond the nurse-patient dynamic, fostering person-to-person connections. "I feel like we are equals. I feel like I am talking to you as a person, or… as one person" (Ms. C).

Being Aware of Our Interactions With Them on a Daily Care

Nurses paid attention to their interactions during routine care, such as morning greetings or temperature checks. "I went there in the morning and took the first step, right? … I would look at the child's facial expression, words, and behavior and decide whether to go now or later" (Ms. F).

Experiencing one's own subjectivity

When nurses interacted with adolescents, they not only valued the adolescents' subjectivity but also remained aware of their own subjectivity. This theme included three subthemes, described below.

Reflecting on My Own Feelings as an Individual

Nurses reflected on and recognized their own emotions, not only as professionals but also as individuals. "Sometimes there is an opportunity to get feedback after they get through it and become adults, and I feel that was okay" (Ms. A).

Feeling Satisfaction in Supporting Their Growth

Nurses derived satisfaction not only from the treatment outcomes of adolescents with cancer but also from witnessing their growth and development. "I think it is a lot of fun with them growing up" (Ms. C).

Experiencing Ease of Involvement by Recognizing the Other Person as a Subject

Nurses expressed that separating themselves emotionally from adolescents and viewing them as subjects made it easier to connect with them. "I think that anxiety and fear are born out of anxiety about whether or not I can do well, or whether or not I can say the right things to them" (Ms. B).

## Discussion

Participants indicated that although they experienced difficulties in interacting with adolescents with cancer, they worked to connect with them based on the adolescents' characteristics and maintained their professionalism as nurses. Prior research revealed that adolescents with cancer sought involvement from healthcare professionals tailored to their generation's unique characteristics, such as their specific knowledge and experiences [14]. Adolescents also required healthcare providers to demonstrate knowledge of cancer treatment, clinical competence, and skills [5]. Therefore, the nurses' attitudes identified in this study aligned with the needs of the AYA population.

This study also highlighted how nurses worked with adolescents with cancer, treating them as individuals rather than merely patients. Adolescents with cancer often face identity-related challenges consistent with Erikson's developmental theory, which describes the conflict between identity and identity diffusion [12]. They experienced further identity upheaval due to their cancer diagnosis [10]. For adolescents to establish their identity, it is important to engage with them as individuals and respect their subjectivity [10]. In addition, because children under 19 years of age are minors, there is a risk that the adolescents' opinions will be ignored. Therefore, these characteristics of adolescents and institutional contexts may have influenced the result that the nurses in this study treated adolescents as individuals rather than merely patients. Furthermore, adolescents valued humor and efforts to build relationships with healthcare professionals, which fostered trust [5]. Therefore, the attitudes of nurses observed in this study were appropriate for the care of adolescents with cancer.

In this study, nurses who interacted with adolescents were "communicating with a sense of distance" and "engaging with an eye to the future." Although these were important in other areas of adolescent medicine, it was thought to be even more important in cancer treatment, where the physical and psychosocial effects have a great impact on their daily life. Even more, the average hospital stay in Japan is longer than in many other countries [11], leading to prolonged interactions between nurses and inpatients. Therefore, it is important to communicate with adolescents, monitoring their conditions in daily care. Additionally, the longer nurses are involved with adolescents, the more opportunities they have to touch adolescents' growth. Furthermore, future implications, such as late complications, are also significant. So, these characteristics of cancer treatment and specific cultural and institutional contexts of Japan may have influenced the subthemes "communicating with a sense of distance" and "engaging with an eye to the future" identified in this study.

The 2022 edition of the Model Core Curriculum for Medical Education states that the content of professionalism is to "keep thinking about unanswerable questions" in Japan [15]. In pediatric oncology nursing, prior research found that experienced nurses relied less on specific coping strategies when experiencing stress and instead found reassurance in the passage of time [16]. Adolescents, who are often in the process of discovering what they value and want to do, are in an inherently unstable state and may experience a loss of autonomy due to their cancer diagnosis. Restoring a sense of control is therefore important [17,18]. Consequently, in adolescent cancer nursing, the ability to remain present with the patient while managing one's own emotional wavering, without rushing to find answers, is of paramount importance. The theme "being with adolescents while managing one's own emotional wavering" identified in this study encapsulated this "keep thinking about unanswerable questions" and "ability to waver." This approach, particularly suited to the AYA generation, deepened the expertise of nurses in this challenging specialty.

Doona et al. described nursing presence as an intersubjective encounter in which nurses viewed patients as unique persons in unique situations and chose to spend time with them while patients invited nurses into their presence [19]. A scoping review of nursing presence in pediatric oncology identified elements such as being present, building therapeutic relationships, and effective communication as hallmarks of nursing presence [20]. Our results revealed that being aware of one's own challenges, maintaining professionalism, and relating to adolescents as individuals all embodied nursing presence. These elements align with previous research and reflect the nursing presence in adolescent cancer care.

Pediatric hematology/oncology remains a challenging and stressful specialty. Spirituality and spiritual well-being (SWB) are essential to sustaining nurses in such environments [21]. SWB encompasses a sense of self and personal understanding. Prior research has revealed that nurses with less than five years of experience in pediatric oncology nursing have higher levels of stress and fewer coping strategies than those with more experience [16]. Early-career nurses in AYA wards often experience emotional burdens even outside work and struggle to detach [22].

In this study, participants had at least five years of experience in adolescent cancer nursing, with half being specialized in pediatric or oncology nursing. Most of them were in their 30s and 40s. The background of the participants may be influenced not only by the inclusion criteria but also by the fact that 50% of nurses in Japan are in their 30s and 40s [23]. These qualifications likely contributed to the participants' ability to reflect on and articulate their nursing care. Therefore, these characteristics of the participants may have influenced the theme of "experiencing one's own subjectivity despite their difficulties" identified in this study. For nurses to continue providing high-quality care in these stressful environments, especially younger staff, it is essential to create opportunities for them to reflect on their own experiences and value both their subjectivity and that of the adolescents. By fostering personal development, nurses can better support adolescents and enhance the quality of care provided.

Implications for nursing clinical improvement

If nurses experience difficulties in interacting with adolescents, they have the opportunity to recognize their own thoughts by talking to others around them, such as staff members in the same ward. Recognizing their own subjectivity through these experiences could reduce the difficulties caused by fluctuation. Therefore, it is necessary to create a team and an environment where individuals feel safe discussing their feelings and thoughts. Additionally, young staff members, especially those with less than five years of experience, are more likely to experience a sense of difficulty and are less aware of their own senses. Therefore, it is important to support them in developing an understanding of their sense of self. Furthermore, the findings of nurses' attitudes and how they deal with difficulties will inform training programs for nurses caring for adolescents with cancer. Self-reflection is suggested as an effective educational program content, and managers are expected to create a work environment in which staff members feel comfortable speaking their minds.

Limitations

This study has several methodological and recruiting limitations. First, most participants belonged to pediatric-only wards or hospitals specializing in children's diseases, and only a few cared for adult patients with cancer. Additionally, the majority were in their 30s to 40s, with only one in their 20s. Therefore, the findings may have been biased. Hence, clarifying the attitudes of nurses working in adult wards and those with less than five years of experience caring for adolescents with cancer is necessary. Second, there is also a selection bias in the request to the facility, as participants were recruited by the head nurse of the department. Our results suggest that nursing presence existed in adolescent oncology nursing. However, it should be evaluated from both sides due to the interactions between the nurse and the patient. Further research should examine the viewpoints of patients, as these alone are insufficient to fully clarify nursing presence in adolescent cancer nursing and identify how adolescents with cancer perceive nurses' care. In the future, these results can be used to develop questionnaire items in quantitative research, such as a Delphi method, for the exploration of educational programs to train the attitudes of nurses caring for adolescents with cancer.

## Conclusions

Semi-structured interviews were conducted with 10 nurses who had at least five years of experience in adolescent oncology nursing to clarify the attitudes of nurses who work with adolescents with cancer. The findings suggest that in adolescent cancer nursing, where nurses tend to experience challenges, it is crucial to remain with adolescents without rushing to provide answers, separate oneself from them, and be present with them while managing emotional wavering. Additionally, mutual subjectivity, where the nurse embraced both the adolescent's and their own subjectivity, was essential. For this reason, it is important to have the opportunity to recognize their own thoughts by talking to others around them and creating a team and an environment that fosters a sense of safety in discussing their feelings and thoughts.
